# 20S immunoproteasomes remove formaldehyde-damaged cytoplasmic proteins suppressing caspase-independent cell death

**DOI:** 10.1038/s41598-017-00757-w

**Published:** 2017-04-05

**Authors:** Sara Ortega-Atienza, Casey Krawic, Lauren Watts, Caitlin McCarthy, Michal W. Luczak, Anatoly Zhitkovich

**Affiliations:** Department of Pathology and Laboratory Medicine, Brown University, Providence, RI 02912 USA

## Abstract

Immunoproteasomes are known for their involvement in antigen presentation. However, their broad tissue presence and other evidence are indicative of nonimmune functions. We examined a role for immunoproteasomes in cellular responses to the endogenous and environmental carcinogen formaldehyde (FA) that binds to cytosolic and nuclear proteins producing proteotoxic stress and genotoxic DNA-histone crosslinks. We found that immunoproteasomes were important for suppression of a caspase-independent cell death and the long-term survival of FA-treated cells. All major genotoxic responses to FA, including replication inhibition and activation of the transcription factor p53 and the apical ATM and ATR kinases, were unaffected by immunoproteasome inactivity. Immunoproteasome inhibition enhanced activation of the cytosolic protein damage sensor HSF1, elevated levels of K48-polyubiquitinated cytoplasmic proteins and increased depletion of unconjugated ubiquitin. We further found that FA induced the disassembly of 26S immunoproteasomes, but not standard 26S proteasomes, releasing the 20S catalytic immunoproteasome. FA-treated cells also had higher amounts of small activators PA28αβ and PA28γ bound to 20S particles. Our findings highlight the significance of nonnuclear damage in FA injury and reveal a major role for immunoproteasomes in elimination of FA-damaged cytoplasmic proteins through ubiquitin-independent proteolysis.

## Introduction

Aging, reactive metabolites and other factors promote formation of misfolded and damaged proteins, which is detrimental to cell functions and is a cause of many neurodegenerative diseases^1^. Proteasome-mediated degradation is the main process for elimination of damaged proteins in human cells^2^. Proteasomes consist of two main components: the catalytic 20S core particle and the 19S regulator particle which together form the 26S proteasome. The 19S complex binds polyubiquitinated proteins, unfolds them using ATP, and feeds the unfolded polypeptide into the interior of the barrel-shaped 20S particle for proteolysis. Small activators such as PA28αβ, PA28γ and PA200 do not require ATP or ubiquitin to stimulate protein degradation by the 20S proteasome^3^. The 20S core particle contains three active subunits with caspase-, trypsin- and chymotrypsin-like activities. These subunits are replaced by related proteases LMP2, LMP10 (MECL1) and LMP7 in immunoproteasomes (i-proteasomes) that are the most abundantly expressed in lymphoid tissues^4, 5^. This exchange of proteolytic activities makes i-proteasomes more efficient at the generation of peptides for antigen presentation^6, 7^. Expression of i-proteasomes in all major tissues and especially in the immunoprivileged sites, such as the retina^8, 9^ and brain^9, 10^, points to i-proteasome functions that are different from antigen presentation. One of these functions can involve responses to protein-damaging conditions, as evidenced by the upregulation of i-proteasomes by nitric oxide^11^ and their role in removal of oxidized proteins^12, 13^. Enhanced abilities of i-proteasomes in degradation of basic proteins are also important for removal of the excess of free histones^14^.

Formaldehyde (FA) is a reactive chemical that is ubiquitously produced in cells during several normal biochemical reactions^15^. These endogenous processes generate biologically significant levels of FA, as evidenced by recent mouse studies in which the loss of a maternal FA detoxification enzyme produced severe growth problems in embryos^16^ and degenerative and genotoxic effects in the tissues of adult animals^17^. The metabolic production of FA is also responsible for neurotoxic effects in victims of methanol poisoning^18, 19^. FA is a common environmental toxicant with many sources of exposure, such as tobacco smoking^20^, off-gassing from consumer goods and emissions by combustion processes^15^. Inhalation FA exposures are linked with higher risks for respiratory^21^ and other cancers^22, 23^. FA carcinogenicity is commonly associated with the formation of genotoxic DNA-protein crosslinks (DPCs) involving histones^15, 21^ due to conjugation of FA with the abundant Lys ε-amino groups within these proteins. Protein damage by FA is quite extensive, as evidenced by a rapid heat shock response and extensive protein polyubiquination^24^. Vulnerability of the nervous system to toxic effects of FA^18, 19, 25^ is consistent with its protein-damaging properties.

Here we examined whether i-proteasomes are involved in responses to FA damage, considering that they display higher activities toward basic proteins^14^. We found that FA triggered the disassembly of 26S i-proteasomes promoting ubiquitin-independent removal of damaged cytoplasmic proteins and suppressing long-term cytotoxic effects. Thus, one of the nonimmune functions of i-proteasomes is protection against proteotoxicity by ubiquitous FA and possibly by other aldehydes. Our findings are also important for the mechanistic understanding of FA toxicities, demonstrating that protein damage outside the nucleus contributes to the development of adverse effects.

## Results

### Gene expression of i-proteasome subunits

**Figure 1:**
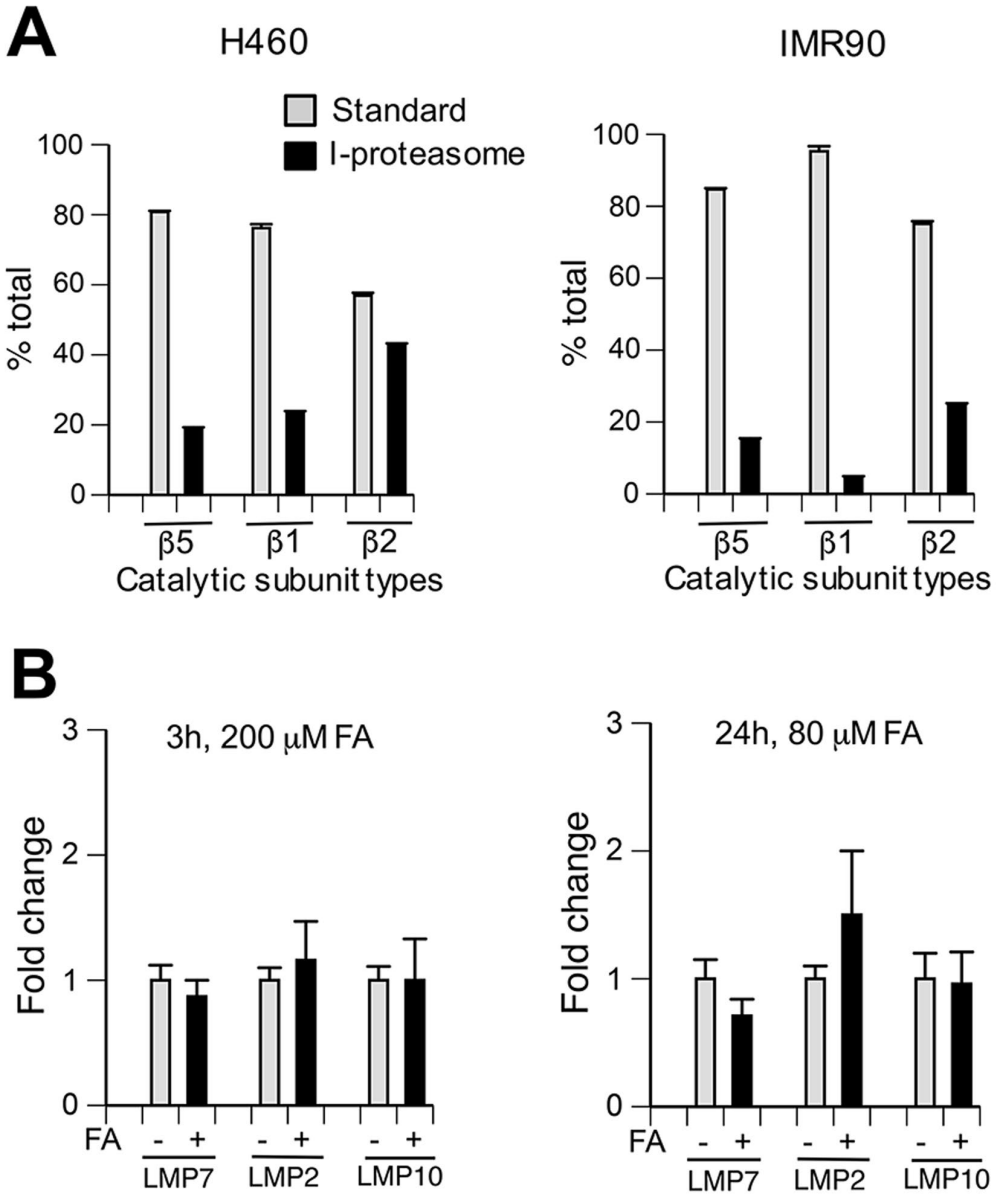
qRT-PCR for catalytic proteasomal subunits. Data are means ± SD for three independent RNA samples. Where not seen, error bars were smaller than symbols. (**A**) Relative amounts of standard and i-proteasomal subunits within three types of catalytic activities (β5: PSMB5 and LMP7, β2: PSMB6 and LMP10, β1: PSMB7 and LMP2). (**B**) Gene expression of i-proteasome subunits is not altered by FA treatments in normal IMR90 cells.

We selected human lung H460 and IMR90 cells as our biological models, which we have used in the past for the characterization of genotoxic signaling and proteotoxicity by FA^24, 26, 27^. Based on gene expression by qRT-PCR, i-proteasomal components constituted 28.7% of all catalytic subunits in H460 cells and 16% in IMR90 cells (Fig. 1A). In both cell lines, LMP7 accounted for approximately 15–20% of β5 subunits with chymotrypsin-like activity, which is the main proteolytic activity of proteasomes. Acute and chronic FA treatments of normal IMR90 cells produced no significant changes in gene expression of i-proteasomal components (Fig. 1B). These negative results were not caused by technical factors, as IMR90 cells showed a strong upregulation of all three i-proteasomal subunits by interferon-γ (4 ng/ml, 24 h: 3.1 ± 0.4, 49 ± 11.4 and 3.7 ± 0.3-fold for LMP7, LMP2 and LMP10 respectively, n = 3).

### FA cytotoxicity

**Figure 2:**
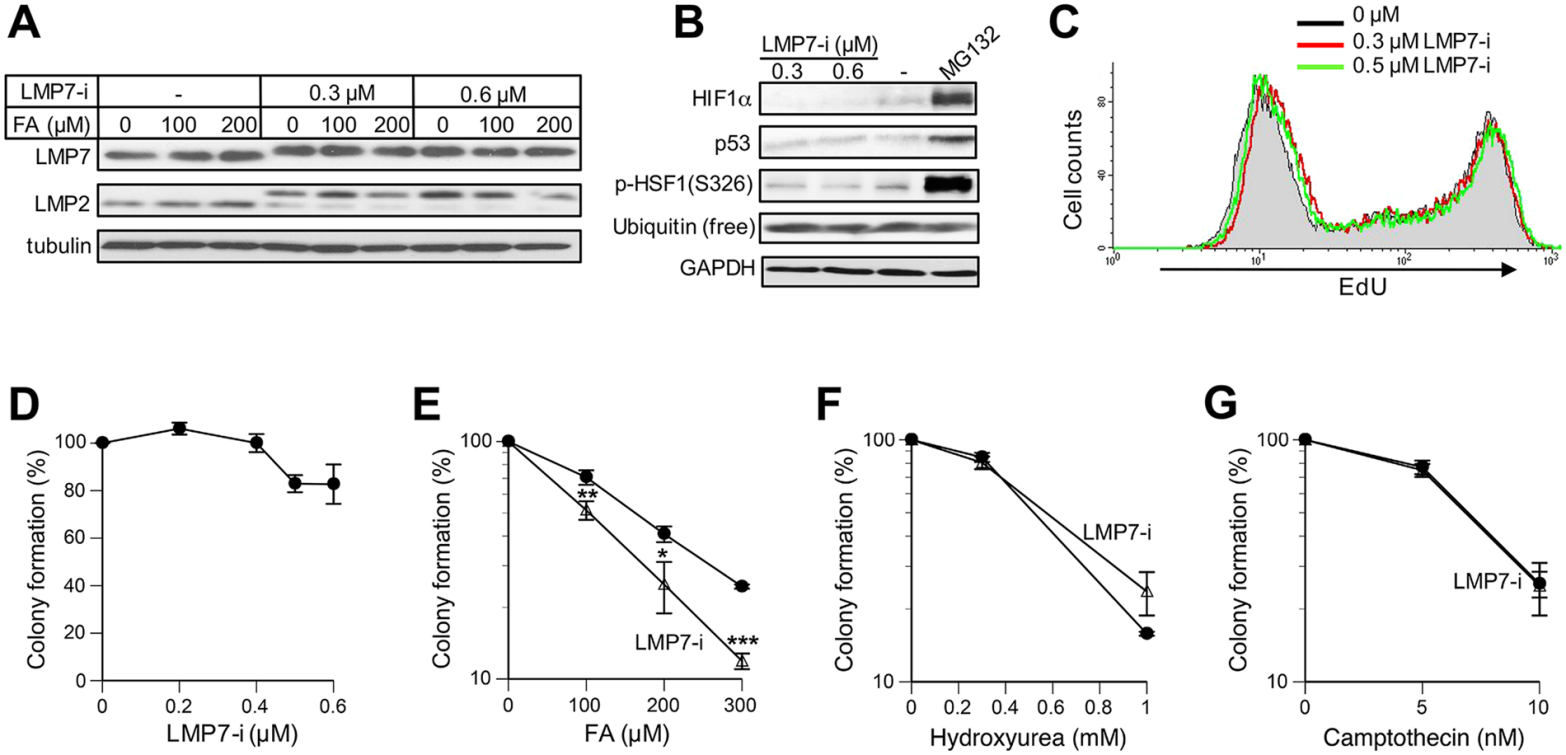
Cytotoxicity of FA and replication stressors in H460 cells. (**A**) Westerns for LMP2 and LMP7 in cells treated with FA and ONX-0914 (LMP7-i) for 3 h. (**B**) Westerns for cells treated with LMP7-i and MG132 (5 μM) for 4 h. (**C**) FACS profiles of EdU incorporation in cells treated with LMP7-i for 6 h. EdU was added during the last hour. (**D**) Clonogenic survival of cells treated with LMP7-i for 24 h. (**E**) Clonogenic survival of cells cotreated with FA and 0.5 μM LMP7-i for 3 h and then incubated for 24 h with 0.5 μM LMP7-i (means ± SD for three experiments in triplicates, *p < 0.05, **p < 0.01, ***p < 0.001). (**F**) Clonogenic survival of cells cotreated for 24 h with 0.5 μM LMP7-i and hydroxyurea or (**F**) camptothecin (means ± SD for two experiments in triplicates).

Mutations in i-proteasome proteins result in specific human disorders, however, knockouts of individual i-proteasome genes in mice did not cause any overt pathophysiological changes^4, 5^, suggesting that the absence of i-proteasome proteins leads to compensatory changes. In agreement with pathological phenotypes of mutated i-proteasomes in humans, pharmacological inhibition of i-proteasomes in mice produced clear physiological effects. A selective inhibitor of LMP7 activity, ONX-0914^28^, is a commonly used tool for interrogation of i-proteasome functions *in vitro* and in animal models of human diseases. Treatments of H460 lung cells with 0.3 or 0.6 μM ONX-0914 (LMP7-i) resulted in the appearance of a slower moving LMP7 band, which was equivalent to a gain of 0.5–1 kDa (Fig. 2A). The magnitude of the observed shift corresponds to a covalent attachment of a single LMP7-i (581 Da molecular weight). LMP2 showed a larger shift (2.5–3 kDa), corresponding to its unprocessed form, which probably results from the mutually dependent incorporation and activation of i-proteasome components^29, 30^. In agreement with qRT-PCR data (Fig. 1B), FA did not alter protein levels of LMP2 or LMP7 (Fig. 2A). To test LMP7-i effects on the constitutive proteasomes, we measured levels of unstable transcription factors HIF1α and p53, which both undergo proteasome-dependent degradation in unstressed cells. In contrast to the proteasome inhibitor MG132, LMP7-i had no effect on the stability of HIF1α and p53 (Fig. 2B). LMP7-i also had no effect on Ser326 phosphorylation of the proteotoxic stress-sensitive transcription factor HSF1, which showed a massive upregulation by MG132. Importantly, the selected dose of our positive control MG132 caused only a partial proteasome inhibition as evidenced by the lack of free ubiquitin depletion (Fig. 2B). The addition of LMP7-i for 6 h produced no changes in cell cycle and DNA replication (Fig. 2C). Longer 24 h incubations with ≥0.5 μM LMP7-i led to modest (15–20%) decreases in the colony formation (Fig. 2D), indicating that i-proteasomes play some role in the normal physiology of H460 cells. The impact of i-proteasomes on FA cytotoxicity was first examined by the clonogenic assay, which integrates all forms of cell death. The presence of LMP7-i during 3 h FA exposures and the subsequent 24 h recovery significantly diminished clonogenic viability of cells (Fig. 2E). Since FA is a potent replication stressor^26, 27^, we also tested cytotoxicity of two other replication stressors hydroxyurea and camptothecin. Hydroxyurea causes stalling of replication forks by depleting cells of dNTPs whereas camptothecin produces DNA-topoisomerase I crosslinks. Unlike FA, cotreatments with LMP7-i and hydroxyurea or camptothecin did not elicit significant changes in clonogenic viability (Fig. 2F,G). Thus, i-proteasomes do not have a general role in cell recovery from replication stress.

### Genotoxic signaling by FA

**Figure 3:**
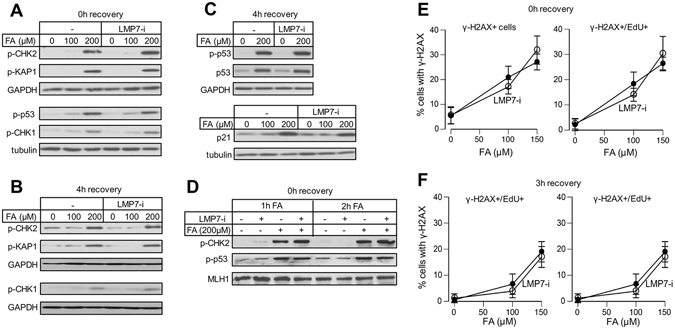
Genotoxic signaling in H460 cells. Cells were treated with FA for 3 h except for panel D. LMP7-i (0.5 μM) was present during and after FA treatments (p-CHK2, p-CHK1, p-KAP1, p-p53: phosphorylated forms). (**A**) Genotoxic signaling at 0 h or (**B**) 4 h recovery after FA exposure. (**C**) Westerns for markers of p53 activation at 4 h recovery post-FA. (**D**) Phosphorylation of CHK2 and p53 in cells treated with FA for 1 or 2 h. (**E**) Percentage of cells containing γ–H2AX foci (γ–H2AX+) and both γ–H2AX foci and EdU staining (γ–H2AX+/EdU+) (means ± SD, n = 3). (**F**) As panel E except that cells were fixed at 3 h post-FA exposure (means ± SD, n = 3).

FA conjugates to lysine-rich basic proteins such as histones and i-proteasomes have higher activity on these types of proteins^14^. Thus, i-proteasomes could be potentially involved in recovery from FA-histone damage that includes DNA-histone crosslinks and FA-histone modification. FA-induced DPCs and chromatin damage triggered activation of the apical kinases ATR^26^ and ATM^27^, respectively. Inhibition of i-proteasomes did not alter phosphorylation of either ATM (CHK2, KAP1) or ATR (CHK1, p53) substrates by FA (Fig. 3A–C). Consistent with the normal activation of p53 (Fig. 3A,C), upregulation of its target, the CDK inhibitor p21, was also unaltered by LMP7-i (Fig. 3C). The presence of LMP7-i during short FA treatments also produced no changes in ATM- or ATR-dependent phosphorylation (Fig. 3D). Ser139-phosphorylated histone H2AX (known as γ-H2AX) is a well-established marker of genotoxic stress^31^. In FA-treated cells, γ-H2AX was found exclusively in S-phase cells and its levels were elevated by inhibition of standard proteasomes^32^. We confirmed that γ-H2AX was present only in the S-phase, as indicated by identical levels of γ-H2AX-positive and γ-H2AX/EdU double-positive cells (Fig. 3E,F). I-proteasome inactivation did not alter the overall or S-phase-specific formation of γ-H2AX.

### Caspase activation and cell death

**Figure 4:**
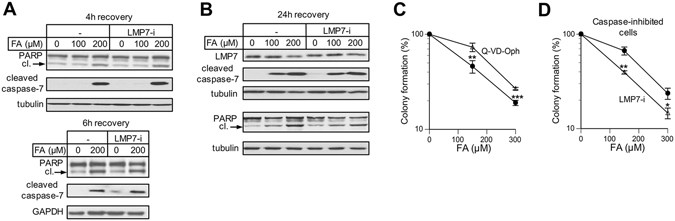
Apoptosis in cells with inactive i-proteasomes. H460 cells were treated with FA for 3 h in the presence or absence of 0.5 μM LMP7-i. The inhibitor was also present during the indicated recovery times. (**A**) Westerns for apoptotic markers at 4 h and 6 h recovery or (**B**) 24 h recovery post-FA treatments (cl.- cleaved PARP). (**C**) Effect of the pancaspase inhibitor Q-VD-Oph (20 μM) on survival of H460 cells. Q-VD-Oph was present during FA treatments and 72 h post-FA (means ± SD, **p < 0.01, ***p < 0.001, n = 3). ((**D**) Impact of LMP7-i on FA toxicity in the presence of the pancaspase inhibitor Q-VD-Oph (20 μM). LMP7-i was coincubated with FA for 3 h and then added for additional 24 h. Data are means ± SD, *p < 0.05, **p < 0.01, n = 3.

FA-treated H460 cells undergo apoptosis, which is in part mediated by the transcription factor p53^26^. We found that the FA-induced production of a caspase-generated PARP fragment and cleaved (active) executioner caspase 7 were not affected by i-proteasome inhibition (Fig. 4A,B). LMP7-i activity was not lost during prolonged incubations, as evidenced by the continuing presence of the slower migrating LMP7 form (Fig. 3B). We next investigated LMP7-i effects on survival of cells with blocked caspase activation. We included a previously validated dose of the pancaspase inhibitor Q-VD-Oph^33^ during FA treatments and the subsequent 72 h recovery and examined the colony formation. As expected based on the detection of activated caspases, Q-VD-Oph significantly decreased clonogenic toxicity of FA (Fig. 4C). We also found that i-proteasome inhibition enhanced clonogenic toxicity of FA even in the presence of Q-VD-Oph (Fig. 4D). These findings together with the normal activation of the proapoptotic transcription factor p53 (Fig. 3A,C) indicate that i-proteasomes are protective against a caspase-independent cell death.

### Stress responses in normal human cells

**Figure 5:**
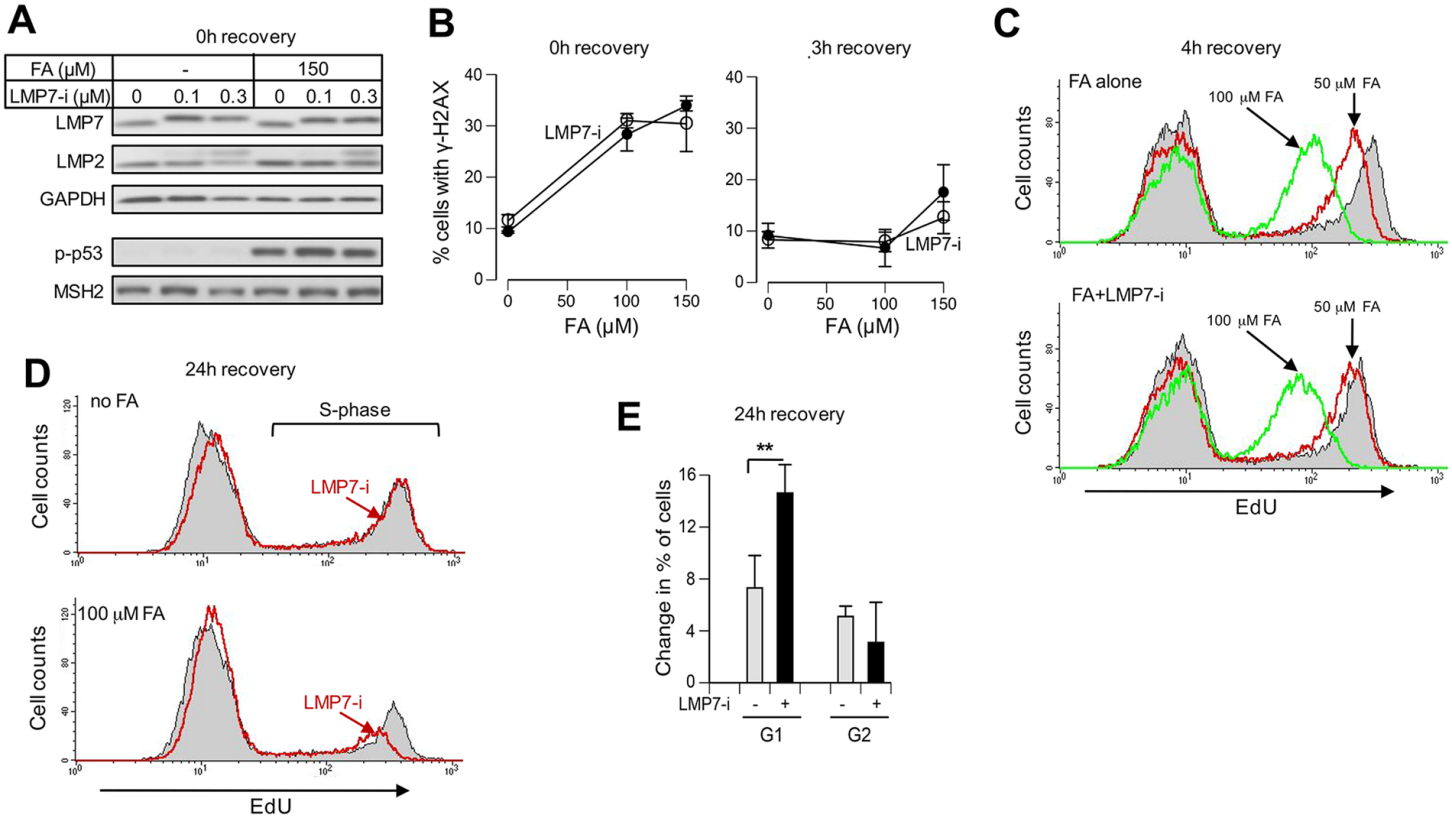
Genotoxic and cell cycle responses in normal human cells. IMR90 cells were treated with FA for 3 h. LMP7-i was present during FA treatments and the subsequent recovery times. EdU was added during the last 1 h of recovery incubations. (**A**) Dose-dependent effects of LMP7-i in cells immediately after FA treatments (p-p53: Ser15-phosphorylated p53). (**B**) Percentage of γ-H2AX-containing cells collected immediately or after 3 h recovery post-FA treatments (n = 3). (**C**) FACS profiles of EdU-labeled cells at 4 h or (**D**) 24 h recovery after FA removal. (**E**) FA-induced changes in G1 and G2 phases. Cells were treated with 100 μM FA for 3 h and collected for FACS after 24 h recovery. Statistics: **p < 0.01, n = 4.

IMR90 normal lung fibroblasts do not undergo apoptosis after moderate FA doses^26^, making them well-suited for cell cycle studies. Inhibition of standard proteasomes in these cells increased early genotoxic signaling responses^27^. The addition of 0.1 or 0.3 μM LMP7-i completely shifted the LMP7 band and the higher dose also produced a slower moving LMP2 band (Fig. 5A). In further experiments, we used 0.3 μM LMP7-i in IMR90 cells. Similar to H460, i-proteasome inhibition in IMR90 cells did not alter early genotoxic responses to FA, such as p53 phosphorylation, γ-H2AX production or DNA synthesis (Fig. 5A–C). However, i-proteasome inactivation impaired a long-term recovery of IMR90 cells from FA damage, resulting in the depletion of S-phase (Fig. 5D) and accumulation of G1 cells (Fig. 5E). Thus, i-proteasomes also play a protective role against FA toxicity in primary human cells.

### HSF1 activation

**Figure 6:**
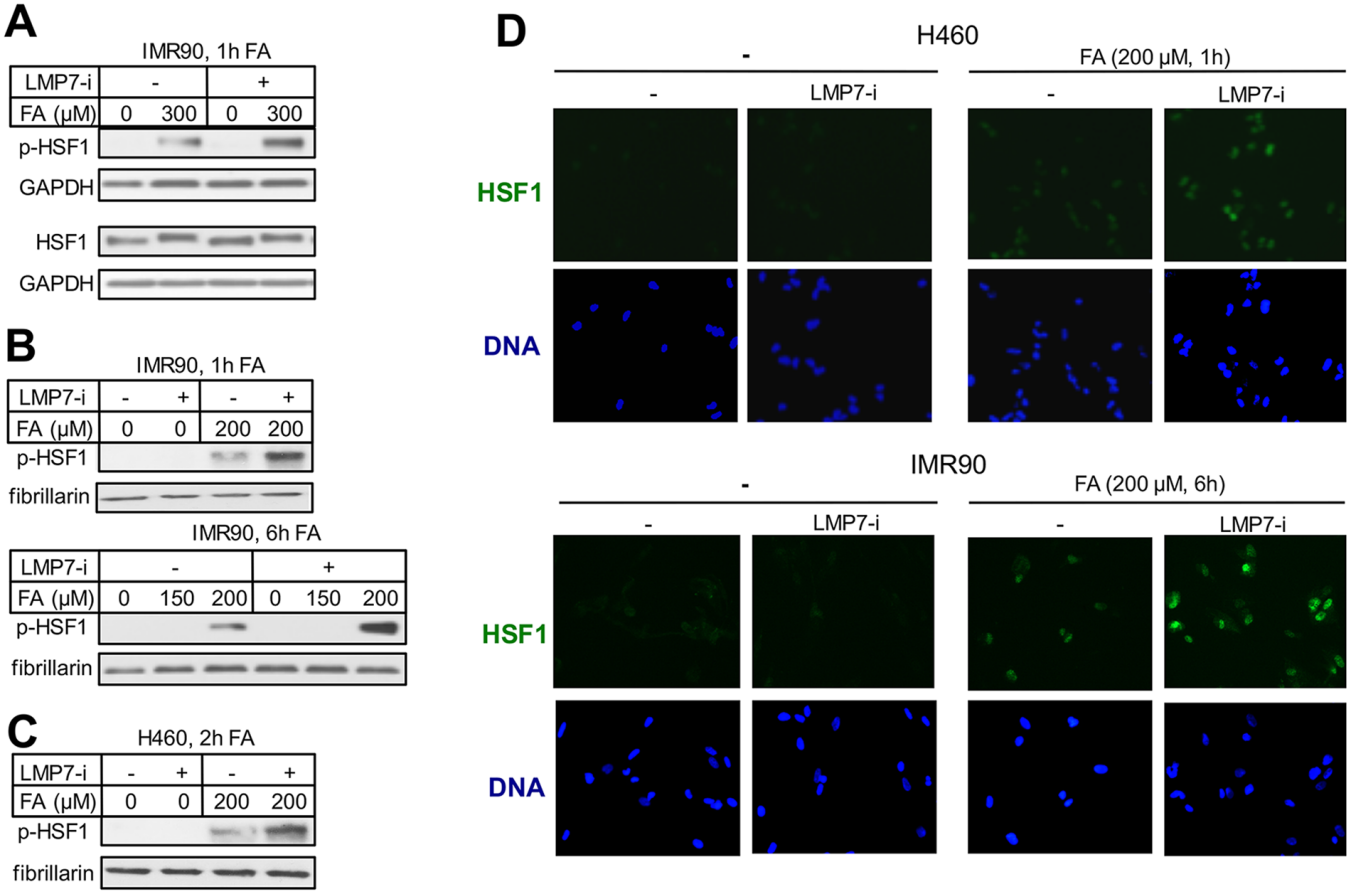
HSF1 activation in cells with inhibited i-proteasomes. Cells were treated with FA in the absence or presence of 0.3 μM LMP7-i. (**A**) Westerns for HSF1 and its S326-phosphorylated form (p-HSF1) in whole cell lysates of IMR90 treated with FA for 1 h. (**B**) S326-phosphorylated HSF1 in the nuclear fraction of IMR90 cells. (**C**) Nuclei-bound phospho-S326-HSF1 in H460 cells treated with FA for 2 h. (**D**) Nuclear HSF1 in FA-treated H460 and IMR90 cells. Cells were extracted with 0.2% Triton X-100 in PBS for 10 min at 4 °C before fixation.

We have recently identified FA as a potent inducer of the heat shock-responsive transcription factor HSF1^24^. HSF1 normally resides in the cytosol but accumulation of misfolded proteins induces its phosphorylation, nuclear translocation and binding to chromatin^34^. We first examined HSF1-Ser326 phosphorylation, which has shown a robust dose-dependent response to FA^24^. I-proteasome inhibition strongly enhanced Ser326 phosphorylation by FA in IMR90 cells whereas HSF1 protein levels remained unchanged (Fig. 6A). A slower mobility of HSF1 in FA samples is typical for proteotoxic conditions and reflects its phosphorylation at multiple sites^34^. FA-treated IMR90 and H460 cells with inactive i-proteasomes also showed higher amounts of nuclear Ser326-phosphorylated (Fig. 6B,C) and total HSF1 (Fig. 6D). Overall, these results indicate elevated levels of FA-induced proteotoxic stress in cells with disabled i-proteasomes.

### Native gel analysis of proteasomes

**Figure 7:**
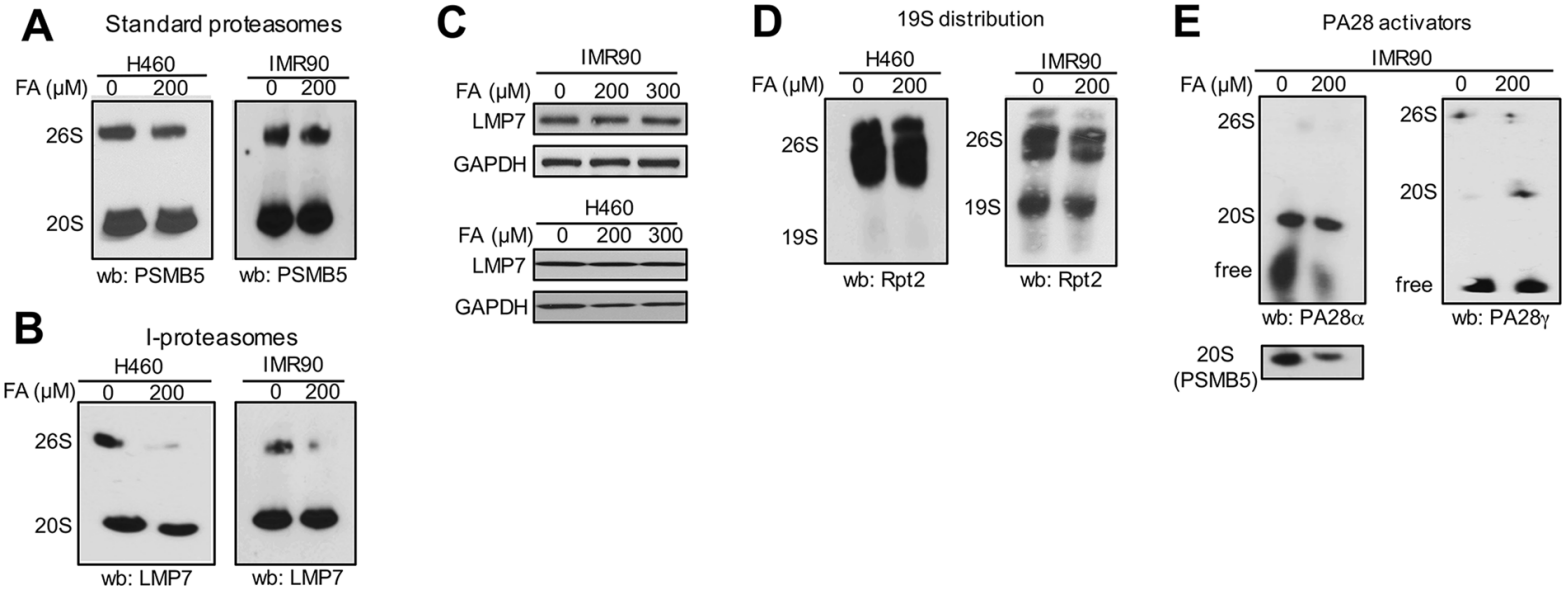
Native gel electrophoresis of proteasomes. H460 and IMR90 cells were treated with 200 μM FA for 2 h. (**A**) Distribution of standard 20S proteasomes between free and 19S-capped (26 S) forms. (**B**) I-proteasome distribution between free (20S) and 19S-capped (26S) forms. (**C**) LMP7 levels in control and FA-treated cells. (**D**) Distribution of 19 S proteasome between its free and 26S-bound forms. (**E**) Proteasome binding of small activators PA28αβ (left panel) and PA28γ (right panel).

A native gel electrophoresis allows separation of 20S and 26S particles that are subsequently identified by western blotting for their specific components^35^. We found that a majority of standard core 20S particles (~65%), detected by the presence of the chymotrypsin-like protease PSMB5, was uncapped in control H460 and IMR90 cells (Fig. 7A). These values are consistent with the results in other mammalian cells^3^. In response to FA, incorporation of regular 20S particles into capped 26S proteasomes slightly decreased in H460 (from 36.6 ± 3.5% to 30.1 ± 1.5%) but remained the same in IMR90 cells (36.1 ± 2.2% for control *versus* 35.5 ± 0.4% for FA). Similar to constitutive 20S proteasomes, a majority of 20S i-proteasomes identified by immunoblotting for LMP7 that replaces PSMB5 in i-proteasomes was also uncapped in control cells (Fig. 7B). Unexpectedly, we found that FA caused severe losses of 26S i-proteasomes in both H460 and IMR90 cells. The overall LMP7 protein amounts were not altered by FA treatments (Fig. 7C). All 19S particles detected by its base component Rpt2 were incorporated into 26S proteasomes irrespective of FA treatments in H460 cells (Fig. 7D). In primary IMR90 cells, 19S proteasomes were present in both free and 26S forms and their distribution was not noticeably altered by FA. Thus, 26S i-proteasomes but not standard 26S proteasomes were sensitive to FA-induced disassembly. Proteolytic activity of 20S core particles is promoted by their association with small PA28 (11S) activators: cytoplasmic PA28αβ and nuclear PA28γ^3^. PA28αβ is particularly important for stimulation of 20S i-proteasomes. We found that FA increased by approximately 2-fold a fraction of PA28αβ that was bound to 20S particles (74.8 ± 2.8% from 36.2 ± 2.8 in controls, n = 2, p = 0.027) (Fig. 7E, left panel). A similar change was found when 20S-bound PA28αβ was normalized to the amount of the 20S component PSMB5 (1.9-fold increase by FA). FA also induced a higher association of PA28γ with 20S particles (Fig. 7E, right panel) although the fraction of 20S-bound PA28γ was lower than that for PA28αβ.

### Ubiquitin reserves and protein polyubiquitination

**Figure 8:**
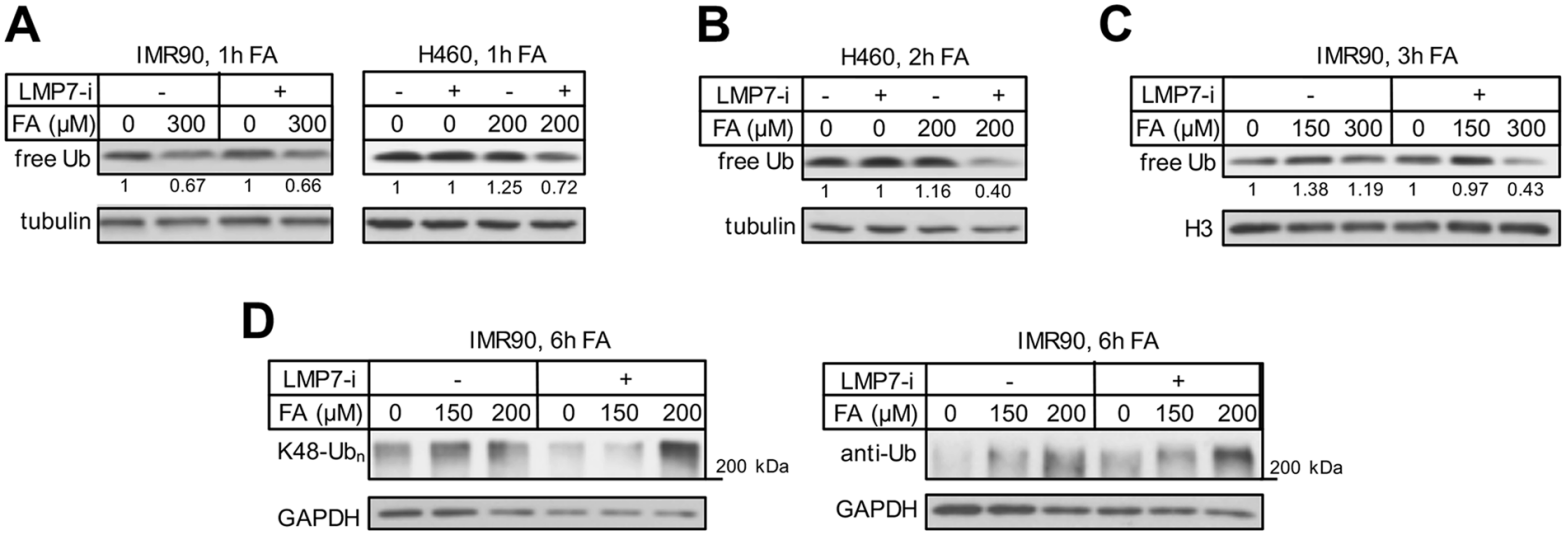
Ubiquitin reserves and protein polyubiquitination. Panels A–C: whole cell lysates were used for westerns and LMP7-i was added at 0.3 μM. (**A**) Free ubiquitin in cells treated with FA for 1 h with and without LMP7-i. (**B**) Free ubiquitin in H460 cells treated with FA+/−LMP7-i for 2 h and (**C**) IMR90 cells treated with FA+/−LMP7-i for 3 h. (**D**) K48-linked (K48-Ub_n_) and overall polyubiquitination (anti-Ub) of cytoplasmic proteins in IMR90 cells treated with FA+/−LMP7-i (0.2 μM) for 6 h.

The disassembly of 26S i-proteasomes and the increased association of PA28 activators with 20S particles indicate a shift towards ubiquitin-independent proteolysis. This raises the question whether cells with inactivated i-proteasomes experience a higher stress on the ubiquitin system due to the need for ubiquitination of additional proteins for destruction by standard 26S proteasomes. One measure of ubiquitin usage is the amount of free ubiquitin^36^. Inhibition of i-proteasomes during 1 h FA treatments produced either no impact (IMR90 cells) or only a modest depletion of free ubiquitin (H460 cells) (Fig. 8A). The inactivity of i-proteasomes during longer FA incubations caused >2-fold depletion of free ubiquitin, which showed no changes in cells with functional i-proteasomes (Fig. 8B,C). Consistent with the dynamics of free ubiquitin, cells with disabled i-proteasomes contained higher levels of polyubiquitinated cytoplasmic proteins as detected with two antibodies (Fig. 8D).

## Discussion

In this work we obtained evidence for the importance of i-proteasome activity in recovery of human cells from injury by carcinogenic FA. Previous studies have found altered FA cytotoxicity in cells with certain DNA repair deficiencies, demonstrating the toxicological significance of DNA damage^37–39^. DPCs are generally considered as the main form of FA-induced DNA damage^15, 21^. DPC repair involves a proteolytic removal of crosslinked proteins, which can be performed by a DNA-dependent protease SPRTN in conjunction with DNA replication^40, 41^. Inhibition of the constitutive proteasomes has also impaired removal of FA-produced DPCs in human cells^42^ and altered genotoxic signaling responses in a manner that was consistent with a diminished repair of DPCs^32^. Histones are the main nuclear proteins modified by FA due to its high reactivity with the side-chain amino group of lysine. Chromatin damage by FA without the involvement of DNA also triggers activation of the genotoxic stress-sensitive kinase ATM^27^. Although i-proteasomes exhibit a higher activity towards basic proteins such as histones^14^, the protective function of i-proteasomes against FA did not result from their involvement in repair of chromatin or DNA. This conclusion is supported by several experimental observations. FA-induced DPCs are potent blockers of DNA replication^26, 27^ due to the inability of the ring-shaped replicative helicase complexes to progress over the steric block imposed by the bulkiness of DPCs^43^. Our results on the normal recovery of DNA synthesis and the normal formation and decay of the genotoxic stress marker γ-H2AX in cells with suppressed i-proteasome activity indicate that the removal of replication-blocking FA-DNA lesions was not affected. The presence of unrepaired DPCs leads to accumulation of cells in the G2 phase, which was not observed in FA-treated cells with inactivated i-proteasomes in contrast to inhibition of standard proteasomes^32^. FA-triggered DNA damage signaling responses include DPC-linked phosphorylation of CHK1 and p53 by ATR^26^ and chromatin damage-induced CHK2 and KAP1 phosphorylation by ATM^27^. None of these signaling responses were altered by i-proteasome inhibition, further strengthening the argument that the prosurvival role of i-proteasomes involved cellular recovery from nongenotoxic injury by FA.

We have recently found that FA induces proteolytic polyubiquitination of proteins throughout the cell, especially in the cytoplasm, and causes a rapid activation of the cytosolic protein damage sensor HSF1^24^. Elevated levels of the activating Ser326 phosphorylation and chromatin binding of HSF1 in cells with inhibited LMP7 indicate that i-proteasome activity was probably most important for removal of FA-damaged cytosolic proteins. This suggestion is further supported by higher amounts of polyubiquitinated cytoplasmic proteins in cells with disabled i-proteasomes. FA caused a near complete release of the catalytic 20S i-proteasome from 26S i-proteasomes but little or no dissociation of standard 26S proteasomes, indicating that i-proteasome activity was shifted from ubiquitin-dependent to ubiquitin-independent proteolysis. Suppression of i-proteasome activity showed a greater usage of ubiquitin in FA-treated cells, pointing to a larger burden of proteins that required ubiquitination. The switch to ubiquitin-independent proteolysis of FA-damaged proteins benefits stressed cells by saving ubiquitin for tagging and proteolysis of other proteins and preserving ATP due to energy independence of 20S-mediated protein degradation. There are no other reports on a stress-induced disassembly of 26S i-proteasomes although the dissociation of standard 26S proteasomes is a known protective response against oxidized proteins^12, 44^. Oxidation of protein cysteines^45^ and the protein chaperone HSP70^46^ have been implicated in the destabilization of standard 26S proteasomes by oxidative stress. It is possible that similar processes are involved in the disassembly of 26S i-proteasomes by FA, which reacts with NH_2_/SH-groups resulting in the formation of damaged/misfolded proteins that can titrate HSP70 away from 26S i-proteasome promoting dissociation of the regulatory 19S particle. The typical substrates for ubiquitin-independent proteolysis in unstressed cells are intrinsically unstable or unstructured proteins^3, 12^, suggesting that these proteins could be particularly vulnerable to misfolding in response to chemical modifications of Lys and Cys by FA. The conformational flexibility of unstructured polypeptides can bring reactive Cys/Lys in a close proximity, allowing FA-induced crosslinking of two amino acids and thereby fixing distorted structures. When not promptly removed, severely misfolded proteins form aggregates that are resistant to proteasome-mediated proteolysis and can exert chronic toxic effects^1^. This pathological mechanism can explain delayed cell cycle abnormalities caused by i-proteasome inhibition in FA-damaged cells.

## Methods

### Chemicals

ONX-0914 (A4011) and Q-VD-Oph (A1901) were from ApexBio. Formaldehyde (F8775), camptothecin (C9911) and hydroxyurea (H8627) were from Sigma. Interferon-γ was purchased from Thermo Scientific (RIFNG50).

### Cells and treatments

H460 and IMR90 cells were purchased from the American Type Culture Collection and cultured as previously described^27^. Cells were treated with FA and other stressors in complete growth media. ONX-0914 (LMP7-i) was added to cells 1 h before FA and present during FA treatments. For induction of i-proteasomes, cells were treated with 4 ng/ml interferon-γ for 24 h.

### Western blotting

Whole cell lysates, cytoplasmic extracts and insoluble nuclear fractions were prepared as recently described^24^. Unless specified otherwise, western blots were run using whole cell lysates. Proteins were separated by SDS-PAGE and immobilized on PVDF membranes. The following primary antibodies were used: LMP2 (sc-37397, Santa Cruz), phospho-S824-KAP1 (A300-767-A, Bethyl); LMP7 (13635), ubiquitin (3933), K48-linked polyubiquitin (5621), HSF1 (4356), PSMB5 (11903), phospho-S317-CHK1 (2344), phospho-T68-CHK2 (2661), phospho-S15-p53 (9284) from Cell Signaling; Rpt2 (ab20239) and phospho-S326-HSF1 (ab76076) from Abcam. Other antibodies were described previously^47^.

### Native gel electrophoresis

Cells were resuspended in a proteasome extraction buffer (50 mM Tris-HCl, pH 8.0, 5 mM MgCl_2_, 0.5 mM EDTA, 1 mM ATP and protease and phosphatase inhibitors) and vortexed for 5 min at 4 °C with acid-washed glass beads (Sigma, G1145). The proteasome-containing supernatants were collected after centrifugation at 15000 g for 15 min, 4 °C. Native electrophoresis was performed on 4% polyacrylamide gels^35^.

### Microscopy

Immunostaining protocols for nuclear HSF1^24^ and phospho-H2AX^48^ have been described earlier. S-phase cells were labeled with 10 µM 5-ethynyl-2′-deoxyuridine (EdU) for 1 h prior to the addition of FA. Primary antibodies were rabbit polyclonal anti-HSF1 (4356S, Cell Signaling) and rabbit polyclonal anti-phospho-histone H2AX (06–570, Millipore). Antibodies were diluted in a PBS solution containing 1% BSA and 0.5% Tween-20 and incubated with cells for 2 h at 37 °C. Images were acquired on the Nikon E-800 Eclipse fluorescent microscope.

### Cell cycle analysis

A recently described procedure was followed^32^. DNA synthesis was measured by EdU labeling (10 µM, 1 h). DNA ploidy was determined by propidium iodide staining (40 µg/ml, 30 min at room temperature). Flow cytometry data were acquired on FACSCalibur (BD Biosciences) and analyzed by the CellQuest Pro software.

### Clonogenic survival

H460 cells were seeded onto 60-mm dishes (400 cells/dish) and treated with chemicals on the next day. After 6–8 days of growth, colonies were fixed with methanol and stained with the Giemsa solution.

### qRT-PCR

Total cellular RNA was purified with TRIzol Reagent (Ambion) and used for reverse transcription reaction (RT First Strand Kit, Qiagen). Real-Time PCR reactions were prepared using the RT SYBR Green ROX qPCR Mastermix and primers from Qiagen and run on the ViiA7 Real-Time PCR System (Applied Biosystems). Expression of i-proteasome subunits after FA treatments was determined by the ΔΔCt method using B2M, GAPDH and TBP mRNAs for normalization. Calculations of the relative gene expression for catalytic subunits within the same type of activity (β1, β2 or β5) included the following steps: 1) determination of the ratio (Ri) of the i-proteasome subunit expression to the standard subunit expression by subtracting the corresponding Ct values and using the resulting number as the power in the binary logarithm and 2) determination of the percentage of the i-proteasome subunit using the following equation: (Ri/1 + Ri) × 100%. The overall percentage of i-proteasomes was calculated by combining individual percentages for β1i, β2i and β5i subunits and dividing by 3.

### Statistics

Two-tailed, unpaired *t*-test was used for the evaluation of statistical differences between the groups.
